# Nicotinamide: A Way to Prevent Fetal Alcohol Syndrome?

**DOI:** 10.1371/journal.pmed.0030147

**Published:** 2006-02-21

The most common cause of nongenetic mental retardation in the Western world is fetal alcohol syndrome (FAS). About one in 1,000 United States children is born with FAS, which is caused by prenatal exposure to alcohol. Children with FAS typically have abnormal facial features and reduced growth. They also have central nervous system abnormalities that lead to impaired learning and memory skills, hyperactivity, and other behavioral problems. These neurological disabilities arise because ethanol disrupts the formation and survival of neurons in the developing brain, particularly in the last trimester of pregnancy and in the first few years of postnatal life when brain development is particularly active.

There is no cure for FAS, but it is 100% preventable. Public health officials recommend that women planning pregnancy and sexually active women who do not use effective birth control avoid alcohol—there is no safe dose of alcohol or safe time to drink it during pregnancy. Sadly, this advice is often ignored. In the US, one in 12 pregnant women admits to drinking alcohol, and one in 30 reports binge drinking (five or more drinks at one time). Given such figures, ways to prevent or attenuate the effects of alcohol on the developing brain are badly needed. Alessandro Ieraci and Daniel Herrera now report that nicotinamide (the amide form of vitamin B3) can prevent some of the deleterious effects of ethanol on developing mice brains, and suggest that nicotinamide might be suitable as a preventative therapy for FAS.

Nicotinamide is the precursor of β-nicotinamide adenine dinucleotide, which enhances the action of many enzymes and is therefore essential for cellular function. Nicotinamide and other forms of vitamin B3 have been used for many years as dietary supplements to treat and prevent pellagra, a vitamin deficiency disease. Large oral doses of nicotinamide have also been used over extended periods of time in clinical trials to treat type I diabetes and bullous pemphigoid (a chronic, autoimmune skin-blistering disease). In addition, recent animal data indicate that nicotinamide is also neuroprotective. In rat models of stroke, for example, it improves neurological outcomes by inhibiting the neuronal apoptosis caused by oxygen deprivation. In apoptosis (a highly organized form of cell death), mitochondrial breakdown releases the protein cytochrome-c, which activates enzymes known as initiator caspases. These activate effector caspases (including caspase-3), which digest other cellular substrates and kill the cell without releasing any potentially harmful molecules. Nicotinamide acts as a neuroprotectant in part by inhibiting cytochrome-c release and caspase-3 activation.

As induction of apoptosis is one known mechanism by which ethanol harms neurons, Ieraci and Herrera asked whether nicotinamide could reduce the effects of ethanol in a mouse model of FAS. Subcutaneous injection of ethanol triggers widespread neurodegeneration in seven-day-old mice—whose brain development is comparable to that of human fetuses in the third trimester of pregnancy. The researchers report that an ethanol injection sufficient to raise blood ethanol levels to those that a human fetus would be exposed to if its mother indulged in binge-like drinking activated caspase-3 and induced the release of cytochrome-c. They then show that nicotinamide injected up to eight hours after the ethanol reduced caspase-3 activation and cytochrome-c release without altering blood or brain ethanol levels.

As the “normalizing” effects of nicotinamide were strongest when it was administered zero to two hours after alcohol exposure, Ieraci and Herrera next investigated whether nicotinamide given two hours after alcohol injection could prevent ethanol-induced neuronal death. To look for early signs of neuronal injury, they stained brain sections with Fluoro-Jade B soon after exposing the mice to ethanol. Most damage occurred in brain regions particularly sensitive to ethanol at this age—namely, the anterior cingulate cortex (which is involved in cognition), the hippocampus (a region needed for learning and memory), and the thalamus (which relays messages from the outside world to other brain regions); nicotinamide treatment reduced this damage. When the researchers stained brain sections for NeuN, a marker of mature neurons, several days after ethanol exposure, they found reduced numbers of neurons (compared with control brains) in similar brain regions, and, again, nicotinamide treatment reduced ethanol's effects. Finally, the researchers used three standard behavioral assays to test whether the reduction in ethanol-induced neuronal death produced by nicotinamide affected the behavior of adult mice. They report that nicotinamide reversed the increase in hyperactivity and the decrease in fear caused by ethanol exposure, and prevented the impairment in learning and memory induced by ethanol.

In mice, then, these results show that nicotinamide can reverse the molecular, cellular, and behavioral effects of ethanol exposure on developing brains. While the beneficial effects observed were most pronounced when nicotinamide was given at the same time or shortly after alcohol exposure, the study suggests that there is a time window of a few hours during which treatment with nicotinamide might be effective. More studies are needed to determine exactly how nicotinamide protects neurons against alcohol-induced damage, but the data raise the possibility that nicotinamide treatment may provide a way to prevent some human cases of FAS.

## 

**Figure:**
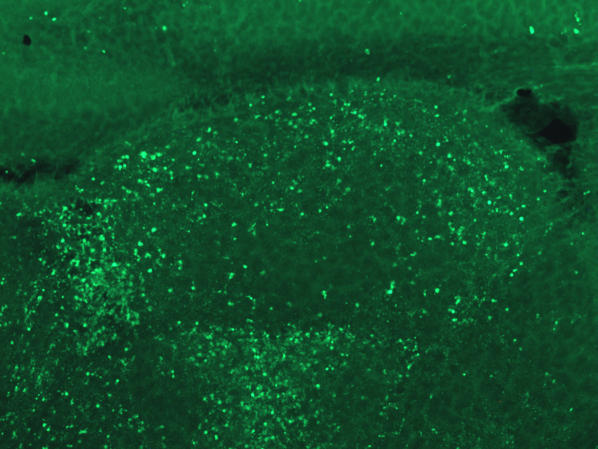
Dying neurons stained with Fluoro-Jade B after ethanol exposure

